# Photooxidation-induced fluorescence amplification system for an ultra-sensitive enzyme-linked immunosorbent assay (ELISA)

**DOI:** 10.1038/s41598-021-85107-7

**Published:** 2021-03-12

**Authors:** Youhee Heo, Kwanwoo Shin, Min Cheol Park, Ji Yoon Kang

**Affiliations:** 1 Center for Biomicrosystems, Brain Research Institute, Korea Institute of Science and Technology, 5 Hwarang-ro, 14-gil, Seongbuk-gu, Seoul, 02792 Republic of Korea; 2 Department of Biomedical Engineering, Sogang University, Seoul, Republic of Korea; 3 Department of Chemistry and Institute of Biological Interfaces, Sogang University, Seoul, Republic of Korea; 4 Absology Co., Ltd., Anyang-si, GyeonGi-Do Republic of Korea; 5 Division of Bio-Medical Science and Technology, University of Science and Technology, Daejeon, Republic of Korea

**Keywords:** Biological techniques, Biotechnology, Chemical biology

## Abstract

This report suggests a method of enhancing the sensitivity of chemifluorescence-based ELISA, using photooxidation-induced fluorescence amplification (PIFA). The PIFA utilized autocatalytic photooxidation of the chemifluorescent substrate, 10-acetyl 3,7-dihydroxyphenoxazine (ADHP, Amplex Red) to amplify the fluorescent product resorufin, initially oxidized by horse radish peroxidase (HRP). As the amplification rate is proportional to the initial level of resorufin, the level of antigen labeled by HRP is quantified by analyzing the profile of fluorescence intensity. The normalized profile was interpolated into an autocatalysis model, and the rate of increase at half-maximum time was quantified by the use of an amplification index (AI). The lower limit of detection, for resorufin or HRP, was less than one-tenth that of the plate reader. It requires only slight modification of the fluorescence reader and is fully compatible with conventional or commercial ELISA. When it is applied to a commercial ELISA kit for the detection of amyloid beta, it is verified that the PIFA assay enhanced the detection sensitivity by more than a factor of 10 and was compatible with a conventional 96-well ELISA assay kit. We anticipate this PIFA assay to be used in research for the detection of low levels of proteins and for the early diagnosis of various diseases with rare protein biomarkers, at ultra-low (pg/mL) concentrations.

## Introduction

The ability to detect biomarker proteins, or nucleic acids, at extremely low concentrations in body fluids is essential to the diagnosis of disease, as well as to research in the life sciences. As the detection of rare biomarkers is crucial for early and accurate diagnosis, there is an urgent need for a highly sensitive assay method^1,2^. Recently, many groups have been searching for disease biomarkers, especially in blood, as liquid biopsy is expected to offer information on the onset and progression of disease, with low cost and high sensitivity^2–6^. Unfortunately, the conventional ELISA (enzyme-linked immunosorbent assay) machines, used in diagnostic laboratories, cannot provide information on rare protein biomarkers with enough sensitivity to distinguish those with disease from healthy controls because the limit of detection (LOD) is usually in the range of tens of pg/mL^4,7–9^.

The previous approaches to overcoming the sensitivity barrier was amplifying the signal to a detectable level. For example, digital ELISA confines fluorescent molecules, generated by enzymes, into reaction chambers of less than 50 fL (femtoliter)^4–6,8,10,11^, and plasmonic ELISA generates colorimetric signals with distinct tonality, as plasmonic nanoparticles are aggregated^12–16^. The bio-barcode technique also enhances sensitivity via DNA-aided signal amplification^17–21^ and ECAS-CIA (enzyme-cascade-amplification strategy-colorimetric immunoassay) allows the detection of low-abundance proteins by coupling with enzyme cascade amplification^22^. However, these approaches are quite complicated and sometimes require high expertise. The digital ELISA, a bead-based immunoassay that can measure atto-molar levels of protein^5,23,24^ requires a large, costly, high-precision machine to be operated by well-trained operators during steps of immunoreaction and washing^25^.

Our study develops a method of photooxidation-induced fluorescence amplification (PIFA) that can amplify a small fluorescence signal to a measurable level. Photooxidation is a chemical reaction where photoexcited species are used to oxidize a substance. ADHP (10-acetyl-3,7-dihydroxyphenoxazine) is a non-fluorescent, colorless compound that is converted to fluorescent resorufin when oxidized by hydrogen peroxide (H_2_O_2_), in the presence of a peroxidase catalyst. It is widely used as a substrate for chemifluorescent detection in ELISA, as well as for the sensitive measurement of extracellular H_2_O_2_^26^. It is reported that resorufin can be reduced by photon and electron donors, and can also be oxidized by peroxidase. These complex behaviors of ADHP and resorufin sometimes complicate the quantitative analysis of fluorescence signals^27–33^.

**Figure 1:**
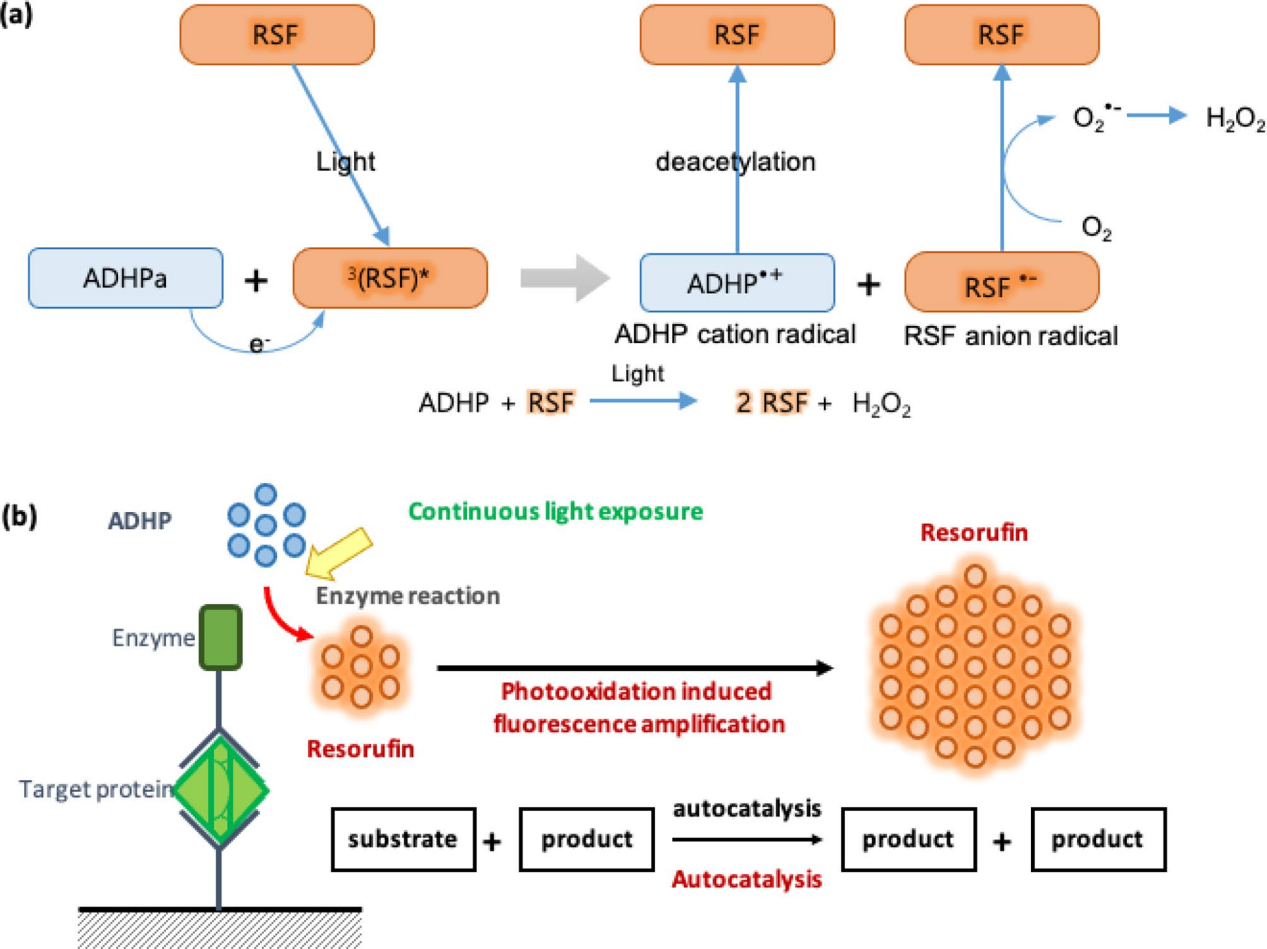
(**a**) Photooxidation of ADHP due to excited resorufin when resorufin in ADHP solution is irradiated by light continuously: ADHP cation radical was deacetylated to resorufin and resorufin anion radical was converted to resorufin. Hydrogen peroxide was generated in photooxidation. (**b**) Sandwich ELISA with PIFA (photooxidation-induced fluorescence amplification): Enzyme labeled at detection, antibody converts ADHP to resorufin at first, and the light irradiation photooxidates the ADHP into resorufin afterwards. The profile analysis quantifies the level of antigen from the rate of increase of fluorescence.

We took advantage of photooxidation as a method for measuring low concentrations of target antigens by the amplification of fluorescence from initial resorufin oxidized by HRP. Continuous light exposure converted the resorufin to triplet-excited state, and ADHP transfers electrons to the resorufin followed by the reformation of resorufin from oxidized ADHP (Fig. 1)^28^. The redox cycling continuously regenerates resorufin exponentially and the fluorescence signal increases until the ADHP is fully consumed. The concentration of initial resorufin is proportional to the increasing rate of fluorescence. Theoretically, a single molecule of resorufin can be detected by PIFA assay, which cannot be accomplished by conventional HRP-based fluorescence assay. The PIFA assay is easy to implement on a conventional sandwich ELISA (Fig. 1b). After immunoreaction of HRP-labeled detection antibody with the antigen, oxidation of ADHP generates resorufin in the presence of HRP and hydrogen peroxide. The mixture of ADHP and resorufin is transferred to a well plate and the continuous measurement of fluorescence is used to track the photooxidation. Meanwhile the fluorescence intensity is recorded and the rate of increase is used to quantify the initial level of resorufin. The information is subsequently used to estimate the level of antigen.

This study evaluates the effect of environmental factors on the rate of fluorescence amplification such as the concentrations of ADHP, hydrogen peroxide, the intensity of light, and the volume of ADHP solution. The enhancement in sensitivity of the PIFA assay was demonstrated by comparing the detection limits of ELISA, between PIFA and a conventional fluorescence detection system. The PIFA is highly compatible with conventional ELISA and provides a simple and low-cost solution for a tenfold increase in sensitivity.

## Materials and methods

### Reagents

ADHP (10-acetyl 3,7-dihydroxyphenoxazine, Amplex Red) was purchased at Invitrogen (Carlsbad, CA, USA). Hydrogen peroxide was obtained from a Quanta Red Enhanced Chemifluorescent HRP Substrate kit (Thermo Scientific, Waltham, MA, USA). Resorufin (424455) and phosphate buffered saline (PBS) was purchased from Sigma Aldrich (St. Louis, MO, USA) and Welgene (Gyeongsangbuk-do, Korea) respectively. PBS of various pH was purchased from the Biosesang (Seongnam-si, Gyeonggi-do, Korea). Biotin-coated plate and HRP-conjugated streptavidin were purchased from Thermo Scientific. Human Aβ_42_ ELISA kits were purchased from Invitrogen. Pooled Human Plasma Apheresis Derived was purchased from Innovative Research (Novi, MI, USA).

### Fluorescence measurement

**Figure 2:**
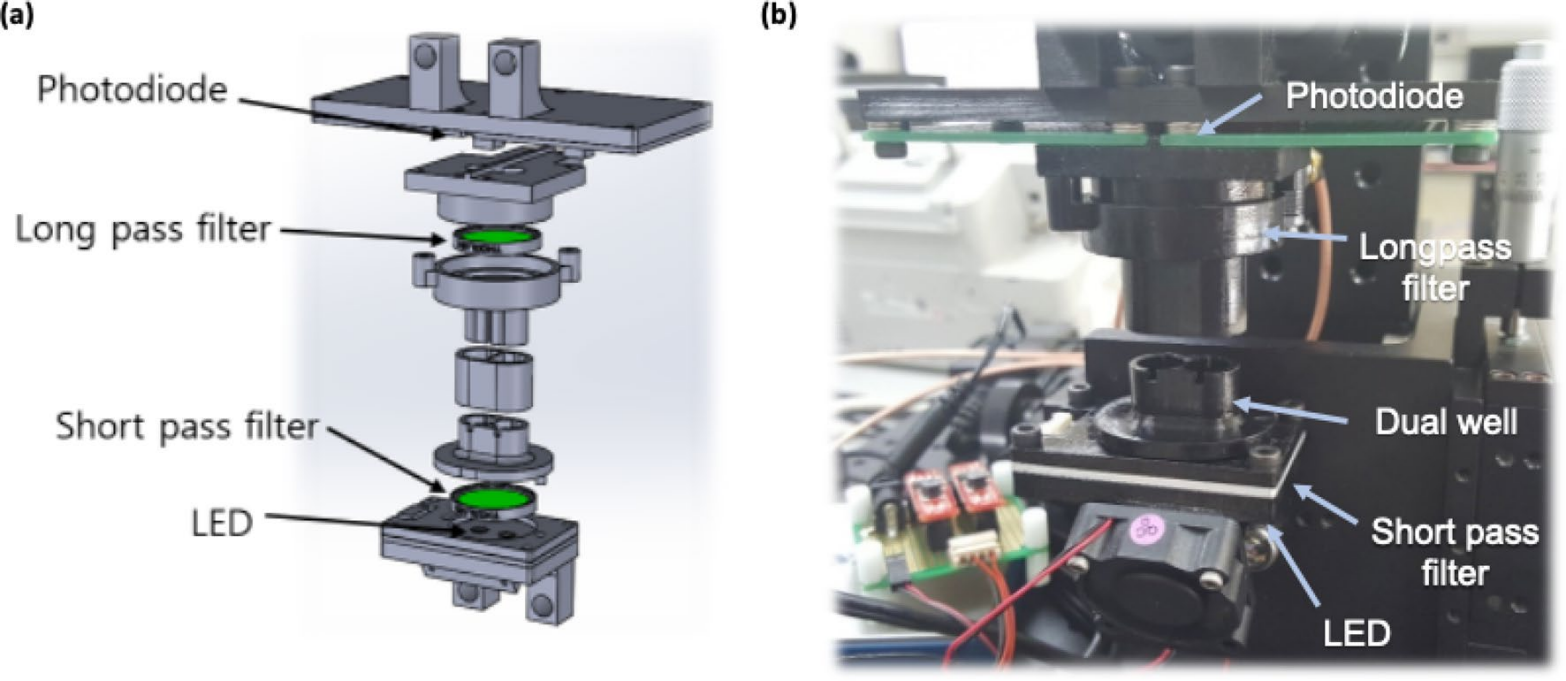
Fluorescence measurement system for PIFA reader (**a**) optical scheme of PIFA reader (**b**) photograph of equipment with dual wells to contain negative control and sample: fluorescence is measured while light is irradiated continuously. LED light from below, through short pass filter was used to excite the fluorescence and emitted light was detected by photodiode at the top.

Fluorescence intensity was continuously measured by in-house vertically aligned optical system, which we called a “PIFA reader”. The incident light from an LED with a short pass filter provided excitation, and the emitted light was measured by a photodiode above the reaction well, through a long pass filter (Fig. 2). The dual wells for a measurement and a negative control were used to calibrate the variation of environmental factors, including light intensity, temperature, moisture, and the initial concentration of resorufin. The wavelength of excitation light for resorufin was 535 nm and that of emission fluorescence was 595 nm. We compared our PIFA reader with a conventional plate reader (BioTek, Winooski, VT, USA).

### Effect of environmental factors on ADHP assay

A solution of 10 mM ADHP was prepared by dissolving 0.26 mg in 100 μL DMSO. It was stored at – 20 °C in a refrigerator and protected from light. The 50 μL of H_2_O_2_ was mixed with the various volumes of ADHP (0.2, 0.4, 0.6, 0.8 and 1 μL) to test the effect of ADHP concentration on the photooxidation. Then PBS was added to the mixture of hydrogen peroxide and ADHP to make the volume of final stock solution 400 μL and the final concentrations of ADHP were 5, 10, 15, 20, and 25 μM. The 90 μL of ADHP stock solution were transferred to one of the dual wells and the fluorescence was measured with the nominal light intensity of 46.8 mW/cm^2^. When investigating the effect of LED light intensity, it was varied between the following values: 24.6, 34.3, 41.3, 46.8, 51.3 and 54.6 mW/cm^2^, as measured by a Compact Power and Energy Meter (PM100D from Thorlabs, Inc., Newton, NJ, USA). The effect of hydrogen peroxide on the rate of increase of fluorescence was also studied as the concentration of H_2_O_2_ was changed from 0 to 0.75% V/V with 1X PBS, at an ADHP concentration of 25 μM. Additionally, the stock solution volume was changed from 40 μL to 120 μL with 10 μL increments. In order to investigate the effect of solution pH, the experiment was conducted by changing the pH of PBS. In the ADHP stock solution, the pH of PBS was changed from 5 to 9.

### Detection of resorufin and HRP with PIFA and plate reader

The diluted resorufin solutions (4 μg/mL to 0.4 pg/mL) were prepared in 1X PBS and stored at 4 °C. The stock solution mixture consisted of ADHP (0.5 μL, 10 mM), H_2_O_2_ (5 μL), and 1X PBS (398.5 μL). This was mixed with the diluted resorufin solutions, at a volume ratio of 1:1, and the final concentrations range of resorufin were from 2 μg/mL to 0.2 pg/mL. The final concentrations of ADHP and hydrogen peroxide were 5 μM and 0.5% V/V, respectively. The 90 μL of solution was transferred to one of the dual well and the fluorescence profile was measured with in-house PIFA reader.

For the test of HRP detection, each well of a biotin-coated 96-well plate was rinsed 3 times with 200 μL of 1X PBS, then filled with 100 μL of streptavidin-HRP solution. The plate was incubated for 30 min at room temperature (RT), followed by washing 3 times with 200 μL of 1X PBS. The same ADHP stock solution as in Sect. 2.3 (100 μL) without resorufin was added and incubated for 15 min, at RT, in a darkroom. The 90 μL solution in the plate well was transferred to the dual well and the intensity of fluorescence was recorded with concentrations of streptavidin-HRP ranging from 125 ng/mL to 0.125 pg/mL.

### ***PIFA assay with human Aβ***_***42***_*** ELISA kit***

The Aβ_42_ standard in the assay kit was serially diluted by a factor of 10, following the protocol in the manufacturer’s manual. A mixed solution of Aβ_42_ standard (50 μL) and anti-Aβ_42_ detection antibody solution (50 μL) was added into each well, and incubated for 3 h at RT, with shaking. After aspiration of the solution, it was washed 4 times with 1X washing buffer. Then anti-rabbit IgG tagged by HRP (100 μL) was added into each well, followed by incubation for 30 min at RT. After the same washing step, ADHP oxidation reaction was carried out for 5, 15, and 30 min at RT in a darkroom by adding 100 μL of ADHP solution. Then, the solution of 90 μL was transferred to one of the dual wells and fluorescence was measured. For the study of sample matrix effect, we substituted the standard diluted solution of Aβ_42_ ELISA kit with pooled plasma (Innovative Research, Mi, USA) to obtain the concentration curve.

### Quantification of PIFA signal

The increment of resorufin concentration is modeled as an autocatalytic chain reaction. Photooxidation of one resorufin (RSF) with ADHP produces two resorufins (Eq. 1).

1 $$RSF+ADHP \leftrightarrows 2RSF$$

**Figure 3:**
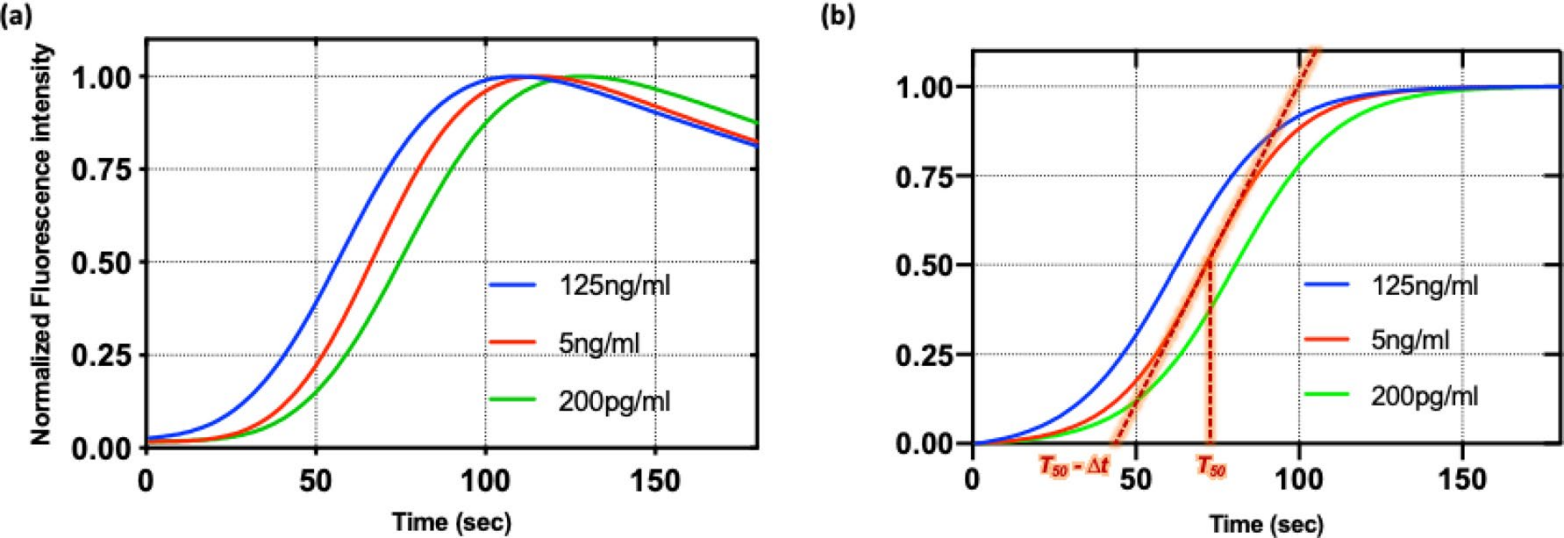
Fluorescence intensity profile when photooxidation amplifies the fluorescence in the mixture of resorufin, ADHP and hydrogen peroxide (**a**) normalized fluorescence intensity profile interpolated with quenching model when initial resorufin concentration was varied (**b**) normalized intensity profile with autocatalysis model whose fitting parameters were extracted from quenching model. Red dotted line is tangential at the half-maximum time, *T*_*50*_*.*

The change of resorufin concentration over time is modeled as a sigmoid curve in Eq. (2).

2 $$RSF(t)= \frac{{ADHP}_{0}+{RSF}_{0}}{1+\frac{{ADHP}_{0}}{{RSF}_{0}} {e}^{-{(ADHP}_{0}+{RSF}_{0})kt}}$$

where *t* is time, *k* is reaction constant, *ADHP* and *RSF* are the concentrations of ADHP and RSF, respectively. *x*_*0*_ is the initial concentration of chemical x. The concentration of resorufin begins at *RSF*_*0*_ and converges to *ADHP*_*0*_ + *RSF*_*0*_. The fluorescence intensity of the solution in the microwell was recorded while LED light irradiated it from below. When measured continuously the fluorescence first increased, but then decreased due to photobleaching of the oxidation product^28^. When the fluorescence intensity profile is normalized to the maximum intensity, the half-maximum time, *T*_*50*_, when the fluorescence intensity approaches 50% of maximum signal, is inversely proportional to the initial level of resorufin (Fig. 3a). The intensity profile did not match perfectly with the sigmoid curve model in Eq. (2) so we modified the fitting model by adding two more factors: background intensity and the quenching rate as in Eq. (3).

3 $$\stackrel{-}{I}\left(t\right)={I}_{BG}+\frac{{A}_{0}+{R}_{0}}{1+\frac{{A}_{0}}{{R}_{0}} {e}^{-{(A}_{0}+{R}_{0})kt}} (1-{D}_{q}t)$$

where $\stackrel{-}{I}$ is normalized fluorescent intensity, ${I}_{BG}$ is background intensity, ${A}_{0}, {R}_{0}, k$ are fitting parameters, and ${D}_{q}$ is quenching rate. The non-linear fitting of intensity profile with Eq. (3) yields background intensity *I*_*BG*_, quenching rate *D*_*q*_, and fitting parameters *A*_*0*_*, R*_*0*_*,* and *k* (Prism 8, GraphPad). The autocatalysis model needs only three parameters, *A*_*0*_*, R*_*0*_*, k*, and is based on Eq. (2). We could retrieve plots of the original model, after removing the offset and quenching effect (Fig. 3b). The analytic solution of half-maximum time is derived from Eq. (2).

4 $${T}_{50}= \frac{\mathrm{ln}{A}_{0 }-\mathrm{ln}{R}_{0 }}{k \left({A}_{0 }+ {R}_{0}\right)}$$

When the initial resorufin concentration is higher, intensity increased more rapidly. The level of resorufin is logarithmically proportional to the half-maximum time. However, this depends on reaction constant *k,* that reflects the environmental factors of the system. Hence, an amplification index (AI) was introduced, to exclude the reaction constant.

5 $$AI= \frac{\Delta t}{{T}_{50}}=\frac{2}{(\mathrm{ln}{A}_{0}-\mathrm{ln}{R}_{0})}$$

AI quantifies the amplification speed at the half-maximum time, and is the point where the time crossed an extended tangential line from the half-maximum time (Fig. 3b). The time difference, *Δt*, indirectly measures the increasing rate and is normalized to half-maximum time. When the initial concentration of ADHP is fixed, AI is almost proportional to the initial concentration of resorufin.

## Results and discussion

### Effect of environmental factors on the photooxidation of ADHP

The photooxidation depends on the reaction constant, *k*, and the concentrations of ADHP and resorufin. The reaction constant is determined by environmental factors, such as temperature, pH, light intensity, wavelength of light, concentration of hydrogen peroxide, and volume of solution. It is also dependent on the oxygen concentration, as well as the concentrations of superoxide dismutase or catalase^28^. The effect of environmental factors on the half-maximum time, *T*_*50*_, were investigated including the concentrations of ADHP and hydrogen peroxide, light intensity, volume of solution and the pH.

**Figure 4:**
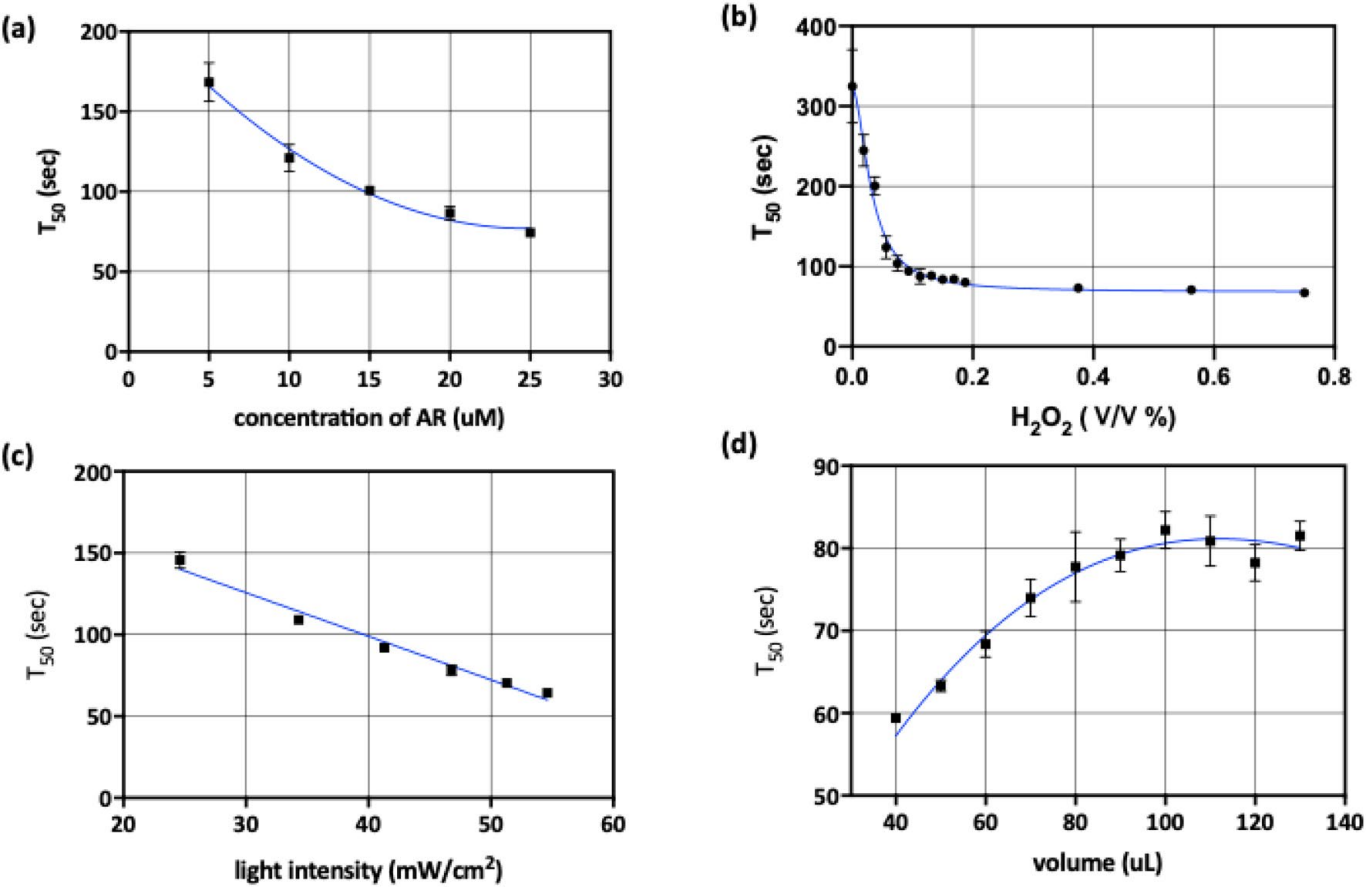
Effect of reaction environmental factors on half-maximum time in PIFA assay (**a**) ADHP concentration (5, 10, 15, 20 and 25 μM) is inversely proportional to half-maximum time (**b**) hydrogen peroxide concentrations (0 ~ 0.75%) is highly nonlinear with half-maximum time and the effect is limited over 0.1% (**c**) light intensity (25 ~ 55 mW/cm^2^) is negatively linear with half-maximum time (**d**) volume of mixture is proportional to half-maximum time and saturated when it is over 90 μL.

When the initial concentration of ADHP, *ADHP*_*0*_, is increased, it is expected to increase the maximum fluorescence intensity, as well as to decrease the *T*_*50*_, from Eqs. (2) and (4). When the ADHP concentration was increased from 5 to 25 μM, the *T*_*50*_ decreased from 170 to 75 s, nonlinearly (Fig. 4a). As the initial concentration of resorufin is much smaller than the concentration of ADHP, the calculated value of *T*_*50*_ in Eq. (4) is almost inversely proportional to ADHP concentration. The experimental result agreed quite well with the autocatalysis model. A higher concentration of ADHP promoted the reaction rate and the fluorescence intensity reached the maximum in a shorter time. However, since the resorufin anion radical consumes oxygen in the process of ADHP photooxidation, a high concentration of ADHP can deplete the concentration of oxygen before the fluorescence reaches its peak. Since the oxygen concentration, in saturation conditions, is usually about 250 μM and the maximum ADHP concentration in our experiment is 25 μM, our experimental results did not show oxygen depletion. If high concentration of ADHP caused oxygen depletion, the addition of catalase could solve the problem by accelerating the dismutation of hydrogen peroxide to water and oxygen.

The photooxidation rate was expected to increase as the concentration of H_2_O_2_ increased due to the decomposition of hydrogen peroxide. When the concentration of H_2_O_2_ was < 0.1%, the half-maximum time decreased linearly, with a steep slope, and when it was > 0.1%, the effect of H_2_O_2_ dramatically lessened, as oxidation of ADHP appeared to be saturated (Fig. 4b). The result showed that the PIFA assay might be useful to detect H_2_O_2,_ without HRP, although the time of measurement exceeds 100 s. When the PIFA assay aims to measure the concentration of resorufin after HRP-based ELISA, the concentration of H_2_O_2_ should be in the range of saturation. When triplet resorufin is converted back to resorufin, the oxygen is consumed to form superoxide, which is consequently dismutated to hydrogen peroxide. If the concentration is in the steep slope region (< 0.1%), the assay result can be significantly changed by the variation in level of H_2_O_2_. In the range of the shallow slope region (> 0.1%), the effect of H_2_O_2_ variation is relatively small.

As the intensity of light is increased, light-initiated oxidation affects the reaction coefficient incrementally. The half-maximum time decreases almost linearly with the increase in light intensity (Fig. 4c). Although resorufin was not added to the ADHP solution, the half-maximum time varies with the change in reaction coefficient, depending on the light energy density. When volume was increased, the time to reach the half-maximum was increased because the light was attenuated, due to absorption by the solution. The diffusion of activated resorufin also takes more time in solutions of larger volume (Fig. 4d). The reaction needs to be propagated from the bottom of the well by diffusion or convection due to temperature differences. When the volume is > 90 μL, the effect of any further increase in volume is limited.

We also considered the effect of pH on the photooxidation (Fig. S1). When the pH of PBS was 5 or 6, the acidic PBS slowed down the amplification of photooxidation because the acidic condition inhibited the decomposition of hydrogen peroxide. On the contrary the alkaline PBS enhanced its decomposition and the half-maximum time was decreased. However, its decrement in alkaline condition was not as much as the increase of acidic condition.

### Enhancement of LOD in the detection of resorufin with PIFA assay

The initial resorufin concentration determines how fast ADHP is converted to fluorescent material by photooxidation. The autocatalysis model, in Eq. (2), is a function of time, and the half-maximum time can be calculated by Eq. (4), and is dependent on the initial resorufin concentration. Experimental results demonstrated that *T*_*50*_ gradually decreased with the concentration of resorufin (Fig. 3b). The initial concentration of resorufin in ADHP solution was nominally zero, but resorufin impurities could be formed during the fabrication process of ADHP or by storage conditions. A negative control solution was used to compensate the resorufin impurities in the ADHP solution. AI’s of both negative control and test sample were measured and the AI ratio of sample to negative control was used as a measure of resorufin concentration in the sample.

6 $$AI ratio= \frac{\mathrm{ln}{A}_{0}- \mathrm{ln}{R}_{NC}}{\mathrm{ln}{A}_{0}- \mathrm{ln}{R}_{0}}$$

**Figure 5:**
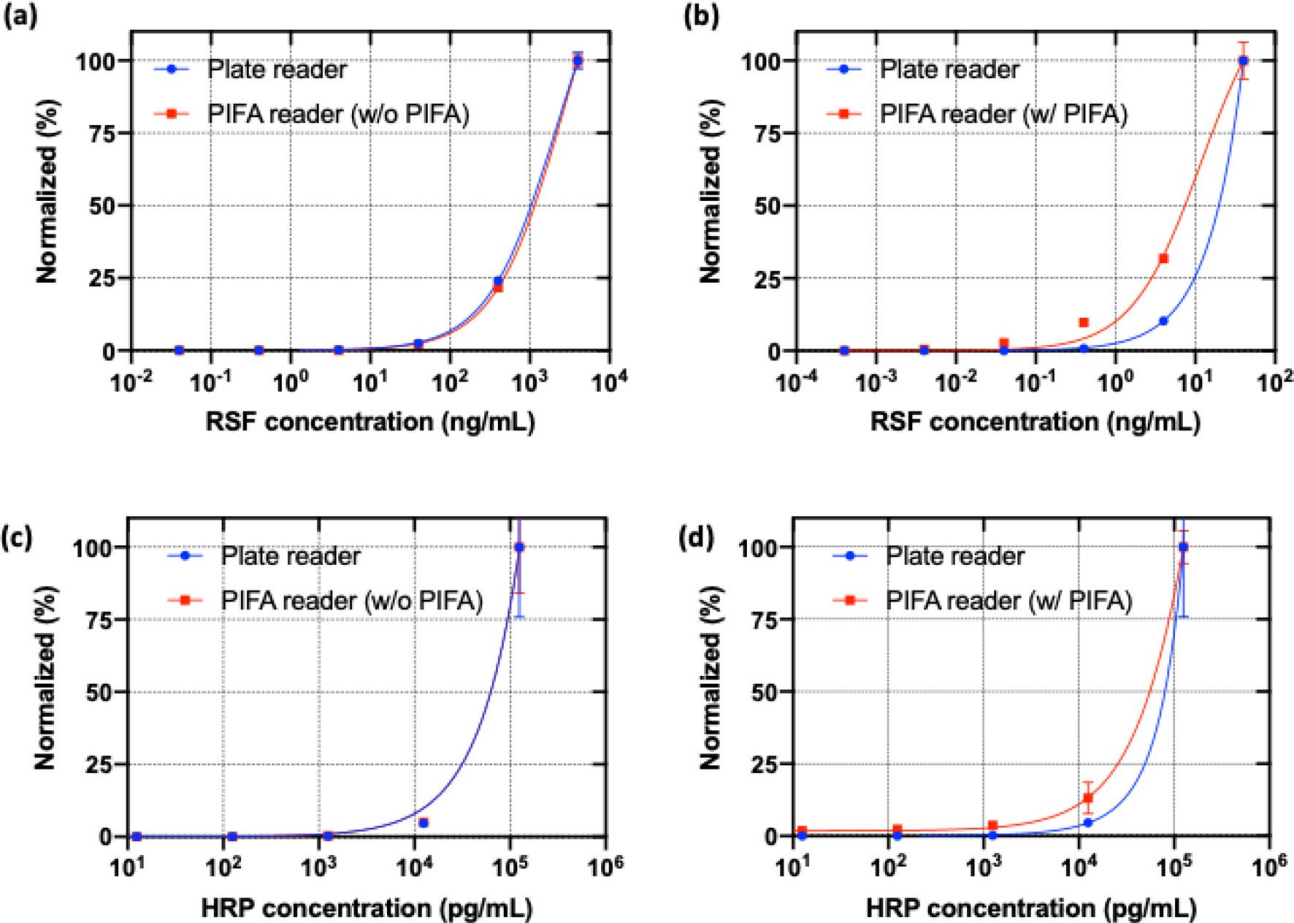
Normalized measurement of PIFA assay and plate reader when concentrations of resorufin (**a**,**b**) and HRP (**c**,**d**) were changed: the measurement output of plate reader is fluorescence intensity and that of PIFA is AI ratio. The limit of detection of two methods were compared after normalization. (**a**,**c**) The normalized fluorescence intensity of resorufin measured by plate reader and PIFA reader with no photooxidation (**b**,**d**) the normalized measurement of plate reader fluorescence and PIFA AI.

When resorufin was diluted from a concentration of 4 μg/mL to 40 pg/mL by 1/10 dilution ratio, the plate reader and PIFA reader showed almost same sensitivity in the detection of resorufin fluorescence (Fig. 5a). When the PIFA assay was implemented, the PIFA reader could measure less than one tenth the minimum threshold concentration (0.4 ng/mL) of the plate reader (Fig. 5b). Continuous fluorescence measurement of resorufin enhanced the LOD by about an order of magnitude, although the fluorescence detection system of the PIFA reader is not as good as that of the plate reader. A simple configuration change in fluorescence measurement successfully promoted the sensitivity of fluorescence detection.

### Enhancement of LOD in measuring HRP with PIFA assay

Hydrogen peroxide, catalyzed by horseradish peroxidase (HRP), reacts with the non-fluorescent substrate ADHP in a 1:1 stoichiometry and generates highly fluorescent product resorufin. We investigated whether the sensitivity of HRP detection could be improved with PIFA assay. The concentrations of HRP to be immobilized on the surface of a 96-well plate was changed in the range of 125 ng/mL to 125 fg/mL by 1/10 dilution ratio in 1X PBS. HRP-conjugated streptavidin (Thermo Scientific, # N100) reacted with the biotinylated surface. The analysis of AI ratio, with the variation of HRP concentration, revealed that PIFA assay was able to measure down to one tenth of the minimum concentration the plate reader could measure (Fig. 5d), although the plate reader and PIFA reader (without PIFA) showed no difference in their lower limit of detection (Fig. 5c). The experimental result of the HRP assay was consistent with that of resorufin detection. It was demonstrated that the HRP with PIFA assay was feasible, and it could be used with a commercial ELISA kit.

### ***Performance enhancement in detection of Aβ***_***42***_*** with commercial ELISA KIT***

**Figure 6:**
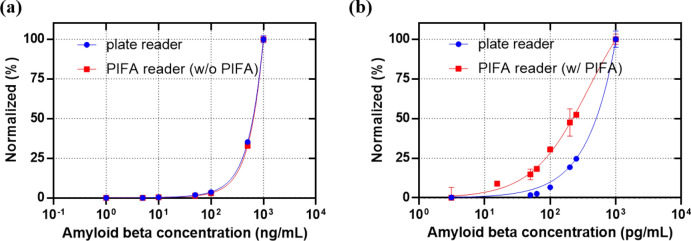
Normalized measurement of PIFA assay and plate reader with the commercial ELISA kit when concentration of amyloid beta was changed: (**a**) the normalized fluorescence intensity of resorufin measured by plate reader and PIFA reader with no photooxidation (**b**) the normalized measurement value of plate reader fluorescence and PIFA AI.

Recent advances in detecting β-amyloid in plasma, using mass spectrometry^34,35^ encouraged the use of ultrasensitive biosensors to measure low levels of Aβ in blood. The PIFA assay was applied to the detection of Aβ_42,_ which is one of the strong biomarker candidates in the detection of Alzheimer’s disease, using a commercial ELISA kit. The standard recombinant Aβ_42_ in the kit was serially diluted (~ 1000 pg/mL) and we followed the manufacturer’s protocol. However, instead of tetramethylbenzidine (TMB), the ADHP solution was added as a substrate for HRP used for labeling of detection antibody. After moving the solution in well plate of ELISA kit to the dual well, the fluorescence was continuously measured by our PIFA reader. When we compared the experimental result of PIFA with that of a plate reader, the threshold for detection of the PIFA assay was tenfold lower than that of the plate reader (Fig. 6). The detection limit of the PIFA assay in the measurement of Aβ_42_ in PBS buffer was in the order of a few pg/mL.

The reaction time of ADHP with HRP enzyme was one of the parameters in optimization of PIFA assay to obtain enough amplification by photooxidation. The times for incubation of other reagents were the same as the protocols in the ELISA kit manual. The short incubation time for enzyme reaction on ADHP could generate a small number of resorufin that could not be enough to differentiate low concentration of the antigen. Longer incubation will form enough resorufin to be photooxidized, but it could take too much time and decrease the throughput of assay. We changed the reaction time to 5, 15, and 30 min after the reaction of anti-amyloid beta 42. As for 5 min incubation, the low enzyme reaction caused measurement error in the low concentration range. In the cases of 15 and 30 min, it seemed that the enzyme reaction generated enough quantity of resorufin for PIFA assay, and both concentration standard curve was quite linear at low concentration range. Hence, we selected 15 min for the enzyme reaction time because 30 min could cause an unnecessary time delay in PIFA assay.

We also investigated the effect of the plasma matrix by spiking standard protein of Aβ_42_. We compared the concentration curves of Aβ_42_ spiked in plasma as well as in standard dilution buffer. The matrix effect on the ELISA of Aβ_42_ was shown in the concentration curve when fluorescence assay was used (Fig. S3a) and also when PIFA assay was applied (Fig. S3b). When PIFA assay was not applied, the fluorescence was measure by a commercial plate reader and it was denoted as “w/o PIFA”. When the plate reader reads the intensity of fluorescence of resorufin, the signals of spiking plasma were less than those of standard protein solution since the abundant proteins of plasma interfered the interaction of antigen and antibody. When PIFA assay was applied, the AI ratio of spiking plasma was less than in high concentrations. In the range of low concentrations, however, the high sensitivity of PIFA generated the AI ratio of spiking plasma that were higher than or equal to stand protein solution, thus demonstrating PIFA assay can detect proteins reliably in plasma sample. When the amyloid beta was spiked in plasma, the limit of detection was about a few tens of pg/mL. The LOD of plasma was one order higher than that of standard dilution buffer. When we applied the PIFA assay, it was observed that the limit of detection was not significantly changed because the slope of signal is still high enough to be detectable in the concentration of pg/mL range. The recovery rates of various concentrations of Aβ_42_ diluted with pooled human plasma ranged from 59 to 152%. At the concentrations higher than 10 pg/mL, they were less than 100% since abundant protein such as albumin interfered the antigen–antibody reaction. At the concentrations lower than 5 pg/mL, the recovery rates higher than 100% might be caused by non-specific interaction of proteins in plasma (Fig. S4A).

There are, however, some points should be remarked in the detection of real samples. The photooxidation of ADHP depends on the initial concentration of resorufin. The oxidation of ADHP into resorufin by light illumination can be disturbed by the concentration of oxidizing agent such as oxygen and hydrogen peroxide. When PIFA is applied to the detection of real sample, the concentration of oxidant should be considered. The concentration of oxygen significantly affects the photooxidation and the low pH lower than 6 could have an effect on the decomposition of hydrogen peroxide. Fortunately, the oxygen concertation in body fluid is enough to support the photooxidation and the pH of most body fluids are also in the range from 6 to 8 except urine sample. If oxidizing agent is carefully considered, it is expected that the PIFA assay is quite robust in the detection of real sample.

## Conclusions

A time history of the PIFA signal was interpolated with the autocatalysis model and the fitting parameters were extracted in the calculation of AI. We examined the effect of environmental factors, including initial ADHP concentration, H_2_O_2_ concentration, light intensity, and reaction volume on the photooxidation of ADHP. Experimental results confirmed that ADHP, H_2_O_2_, and intensity of light promoted photooxidation, while the reaction volume decreased photooxidation. The factors of ADHP concentration, light intensity, and reaction volume affect the half-maximum time almost linearly. However, the concentration of hydrogen peroxide affects the half-maximum time nonlinearly. In the region of high concentration, it seemed that the effect of H_2_O_2_ was saturated_._ When the PIFA assay was applied to measure the concentration of resorufin and HRP, the LOD was enhanced about tenfold compared with a commercial plate reader. We also demonstrated that the PIFA assay could be applied to a commercial ELISA kit for detection of Aβ_42_. It decreased the LOD more than tenfold and could improve the performance of conventional ELISA kit.

The quantification of the profile of PIFA could be used to detect the level of a protein biomarker at concentrations of a few pg/mL. The principle of PIFA is almost the same as the quantification of real-time PCR (polymerase chain reaction) if the light irradiation is controlled. The amplification of fluorescence or color by light irradiation seems to be a useful method for assay enhancement. Another candidate is the colorimetric substrate, o-phenylenediamine dihydrochloride (oPD), widely used as a substrate for ELISA. It is also a photosensitizer and the amplification of the signal by photons is widely known. PIFA assay seems one of promising signal amplification methods to detect low levels of a protein. We believe that PIFA assay can be used to measure rare protein biomarkers in blood samples, such as amyloid beta and cardiac troponin, for the early detection of disease.

## Supplementary information

Supplementary information.
