# Supplementary material for: Photooxidation-induced fluorescence amplification system for an ultra-sensitive enzyme-linked immunosorbent assay (ELISA)
Source: Sci Rep. 2021 Mar 12;11:5831. doi: 10.1038/s41598-021-85107-7 (PMC7954804; doi:10.1038/s41598-021-85107-7)
Supplement: Supplementary file 1 — Supplementary information. [file 41598_2021_85107_MOESM1_ESM.docx]

Supporting Information

Photooxidation-induced fluorescence amplification system for an ultra-sensitive enzyme-linked immunosorbent assay (ELISA)

*Youhee Heo, Kwanwoo Shin, Min Cheol Park^*^, and Ji Yoon Kang^*^*

^1^ Center for BioMicrosystems, Brain Science Institute, Korea Institute of Science and Technology, Seoul, Republic of Korea

^2^ Department of Biomedical Engineering, Sogang University, Seoul, Republic of Korea

^3^ Department of Chemistry and Institute of Biological Interfaces, Sogang University, Seoul, Republic of Korea

^4^ Absology Co., Ltd.

^5^Division of Bio-Medical Science and Technology, University of Science and Technology, Daejeon, Republic of Korea


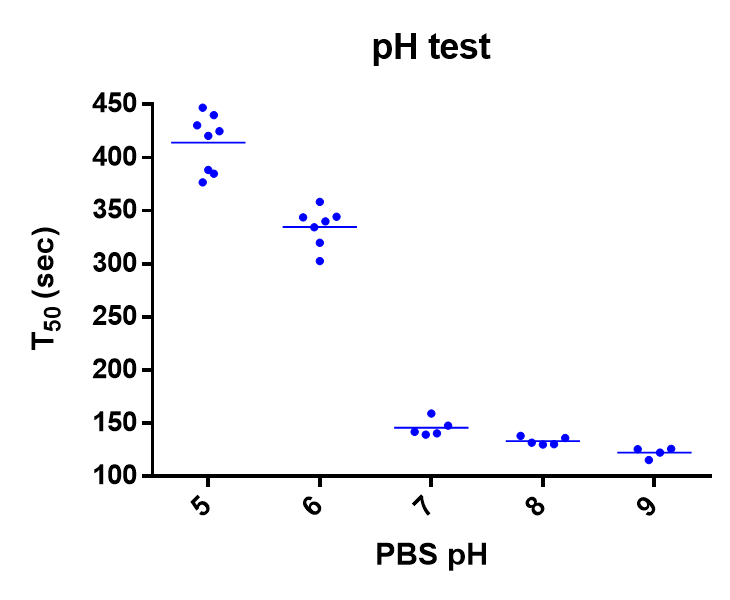


Figure S1. Effect of pH on the half-maximum time


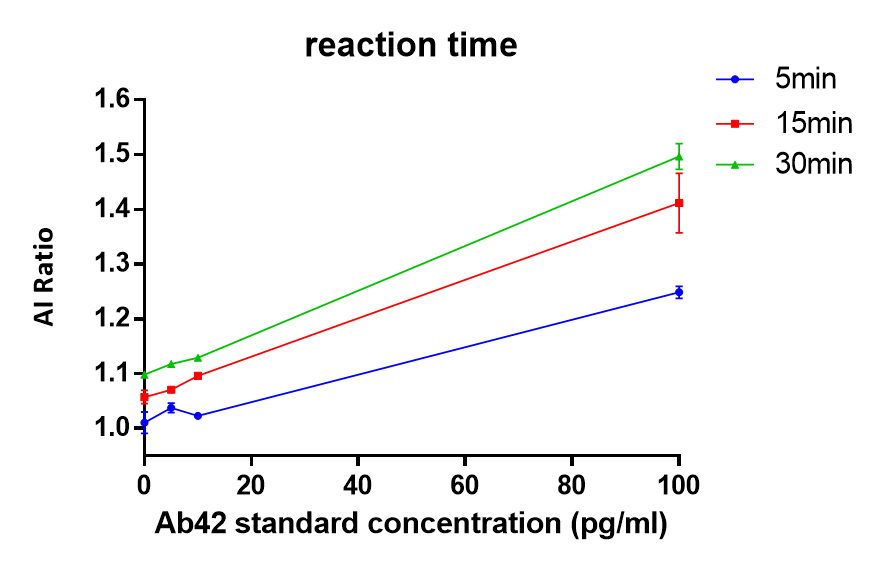


Figure S2. Effect of reaction time on the accuracy of PIFA-based ELISA


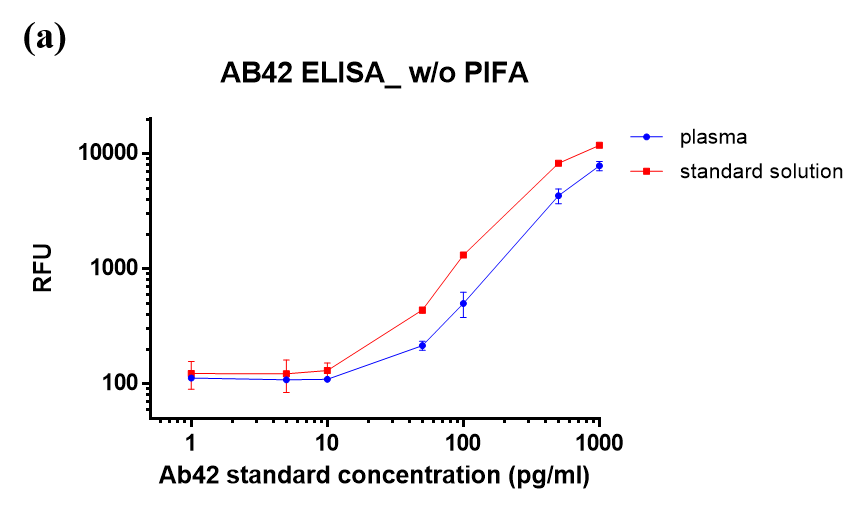


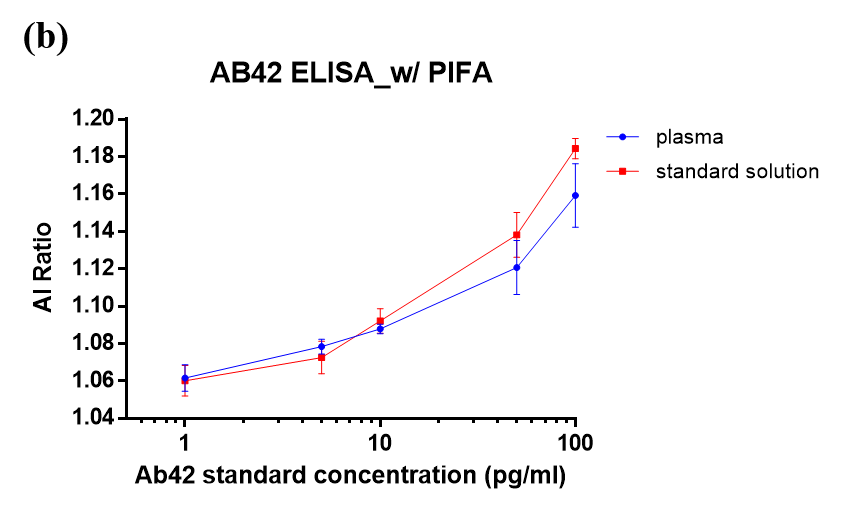


**Figure S3. Matrix effect for ELISA. (a) w/o PIFA (b) w/ PIFA**


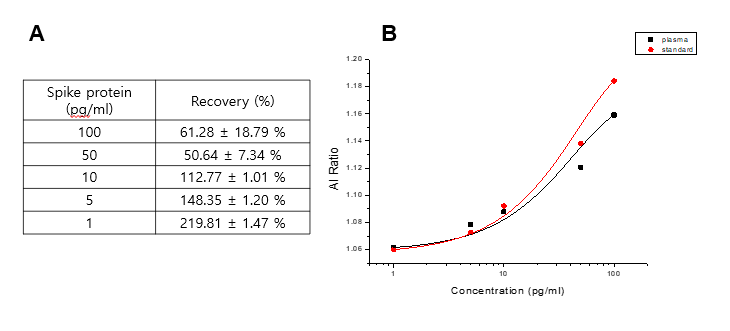


**Figure S4. Recovery rate for ELISA w/ PIFA.**
